# Nailfold Microvascular Abnormalities Are Associated with the Standardized In-Hospital Blood-Pressure Phenotype and Carotid Wall Thickness in Treated Hypertension: An Integrated Vascular Phenotyping Study

**DOI:** 10.3390/jcm15187097

**Published:** 2026-09-13

**Authors:** Marius Badalica-Petrescu, Adrian-Cosmin Ilie, Codruta Craciun, Emilia Elena Clej, Alina Andreea Tischer, Ionut Capraru, Ovidiu Calin Ilie

**Affiliations:** 1 Department of Cardiology, “Victor Babeș” University of Medicine and Pharmacy, Eftimie Murgu Square 2, 300041 Timișoara, Romania; marius.badalica-petrescu@umft.ro; 2 Department of Public Health and Sanitary Management, “Victor Babeș” University of Medicine and Pharmacy, Eftimie Murgu Square 2, 300041 Timișoara, Romania; ilie.adrian@umft.ro; 3 Doctoral School, Department of General Medicine, “Victor Babeș” University of Medicine and Pharmacy, Eftimie Murgu Square 2, 300041 Timișoara, Romania; codruta.craciun@umft.ro (C.C.); emilia.clej@umft.ro (E.E.C.); 4 Ear-Nose-Throat Department, “Victor Babeș” University of Medicine and Pharmacy, Eftimie Murgu Square 2, 300041 Timișoara, Romania; 5 Department of Epidemiology, “Victor Babeș” University of Medicine and Pharmacy, Eftimie Murgu Square 2, 300041 Timișoara, Romania; 6 Discipline of Dental Prostheses Technology (Dental Technology), Department I, Faculty of Dental Medicine, “Victor Babeș” University of Medicine and Pharmacy, Eftimie Murgu Square 2, 300041 Timișoara, Romania; ilie.ovidiu@umft.ro

**Keywords:** arterial hypertension, nailfold capillaroscopy, microvascular rarefaction, carotid intima–media thickness, endothelial dysfunction, arterial stiffness, hypertension-mediated organ damage

## Abstract

**Background/Objectives**: Hypertension damages the microcirculation, endothelium, and large arteries, yet these domains are rarely assessed together. We examined whether nailfold microvascular abnormalities track the standardized in-hospital blood-pressure phenotype and carotid wall thickness. **Methods**: Cross-sectional secondary analysis of 217 treated hypertensive adults hospitalized July 2025–June 2026. Phenotyping included nailfold capillaroscopy, flow-mediated dilation, reactive hyperemia index, pulse wave velocity, and carotid ultrasonography. A prespecified exploratory 0–10 microvascular score summarized rarefaction, impaired flow, and morphological abnormalities. Outcomes were the uncontrolled standardized in-hospital BP phenotype (SBP ≥ 140 and/or DBP ≥ 90 mmHg) and mean carotid intima–media thickness (cIMT). **Results**: Of 217 patients (mean age 61.3 ± 9.9 years; 50.7% men), 145 (66.8%) met the uncontrolled standardized in-hospital BP phenotype. The score was higher in that group (median 3 [IQR 2–4] vs. 2 [1–3], *p* < 0.001) and correlated with cIMT (Spearman ρ = 0.536; FDR-adjusted *p* < 0.001). Each point was associated with higher adjusted odds of the uncontrolled standardized in-hospital BP phenotype (OR 1.70, 95% CI 1.31–2.20; *p* < 0.001) and 0.0076 mm higher cIMT (95% CI 0.0004–0.0147; *p* = 0.040), attenuated in the expanded sensitivity model (*p* = 0.138). **Conclusions**: Greater nailfold microvascular abnormality burden was associated with the uncontrolled standardized in-hospital BP phenotype and an adverse multidomain vascular phenotype. The cIMT association was modest and adjustment-sensitive. These cross-sectional findings concern a protocolized in-hospital BP measurement session and do not establish 24 h, home, or long-term BP control or distinguish sustained uncontrolled hypertension from white-coat or masked uncontrolled BP patterns. Prospective external validation is required.

## 1. Introduction

Arterial hypertension remains a dominant modifiable contributor to cardiovascular, cerebrovascular, and renal morbidity. Contemporary European guidance increasingly emphasizes total cardiovascular risk and hypertension-mediated organ damage (HMOD), rather than treating office blood pressure as an isolated numerical target [1,2]. Despite major therapeutic advances, population-level awareness, treatment, and control remain heterogeneous, and residual vascular risk is substantial even among treated patients [3,4]. This residual risk is biologically plausible because cumulative pressure exposure acts on multiple vascular territories over years, whereas a clinic blood-pressure measurement captures only a brief segment of that exposure.

The hypertensive vascular phenotype is not confined to conduit arteries. Increased peripheral resistance is generated predominantly within small arteries, arterioles, and the microcirculation. Human physiological studies have shown impaired microvascular dilatation and reduced capillary density in individuals predisposed to high blood pressure, supporting the possibility that microvascular abnormalities may precede or accompany established hypertension [5]. Both functional non-perfusion and structural rarefaction have subsequently been demonstrated in essential hypertension [6,7,8]. Importantly, capillary rarefaction may persist despite antihypertensive treatment and acceptable clinic blood pressure, suggesting that normalization of brachial pressure does not necessarily equate to restoration of microvascular architecture [9,10].

Nailfold capillaroscopy provides direct, non-invasive visualization of the human microcirculation. Beyond capillary density, it can characterize red-blood-cell velocity, loop dimensions, tortuosity, dilation, microhemorrhages, avascular areas, and ramified capillaries. Although capillaroscopy is best standardized in rheumatology, observational work has also identified non-specific capillaroscopic abnormalities in hypertension and other cardiometabolic conditions [11,12]. A recent cardiovascular-focused review highlighted the potential of nailfold videocapillaroscopy for peripheral microangiopathy assessment while emphasizing the need for harmonized methodology and outcome validation [13].

At the macrovascular level, carotid intima–media thickness (cIMT) is a well-established ultrasound measure of arterial wall morphology. Its interpretation requires nuance: cIMT is influenced by age, blood-pressure exposure, smooth-muscle hypertrophy, and atherosclerotic processes and should not be considered synonymous with focal carotid plaque [14,15]. Nevertheless, continuous cIMT has repeatedly been associated with future cardiovascular risk, and standardized carotid imaging remains highly informative for mechanistic and epidemiologic vascular research [16,17,18].

A central unresolved question is whether an easily visualized peripheral microvascular phenotype mirrors structural and functional abnormalities in other vascular beds among patients with established hypertension. Demonstrating concordance across nailfold capillaries, endothelial function, large-artery stiffness, renal microvascular injury, and carotid wall morphology would support a systemic vascular-injury construct rather than isolated organ-specific abnormalities. Accordingly, the primary objective of this study was to determine whether nailfold microvascular abnormality burden was associated with the operationally defined standardized in-hospital blood-pressure phenotype in treated adults. The principal structural vascular objective was to quantify the relationship between the microvascular phenotype and mean cIMT. Secondary objectives were to examine relationships with endothelial function, arterial stiffness, albuminuria, renal function, and cognitive performance. We hypothesized that greater microvascular abnormality burden would be associated with the uncontrolled standardized in-hospital BP phenotype and a more adverse multidomain vascular phenotype.

## 2. Materials and Methods

### 2.1. Study Design, Setting, and Participants

This was a single-center cross-sectional secondary analysis of clinical and vascular phenotyping data acquired during hospitalization and analyzed retrospectively in the Internal Medicine II Department of the Railway Clinical Hospital Timișoara (Spitalul Clinic C.F. Timișoara), Timișoara, Romania. The analytic cohort comprised 217 adults with established arterial hypertension hospitalized between 1 July 2025 and 30 June 2026 who completed the vascular, laboratory, and cognitive assessment bundle represented in the study database. The available database was a fully phenotyped analytic dataset rather than a screening registry: it did not retain the denominator of all hypertensive admissions considered for extended phenotyping or detailed reasons for noncompletion. Consequently, the cohort is not presented as a consecutive population-based series. Within the 217 included records, all variables analyzed in this report were complete and no imputation was required. The study is reported in accordance with the STROBE recommendations for observational research [19]. The same institutional cohort has also been used for a separate, non-overlapping research question concerning screening-level cognitive performance, reported in a companion manuscript; the present analysis addresses microvascular–macrovascular concordance, uses different outcomes and models, and reproduces no results from that report. The analytic dataset did not contain a standardized variable coding the principal reason for hospitalization; therefore, a reliable distribution of admission diagnoses could not be reconstructed for this secondary analysis.

Eligibility required age ≥ 18 years, a documented diagnosis of arterial hypertension established before admission, and completion of the vascular, laboratory, and cognitive assessment set used in the present analysis. Patients were excluded if any component of the vascular-phenotyping protocol was missing or technically inadequate, if the clinical condition at admission precluded standardized assessment (hemodynamic instability, acute coronary syndrome, acute stroke, acute infection, or decompensated heart failure), or if they had a condition capable of producing disease-specific capillaroscopic abnormalities, namely systemic sclerosis or another connective-tissue disease, Raynaud phenomenon, or local finger trauma or infection at the site of examination. Patients unable to provide informed consent or to complete cognitive testing because of severe sensory impairment or a previously established dementia diagnosis were also excluded. These criteria define a complete-phenotyping analytic cohort and should not be interpreted as evidence that every otherwise eligible hypertensive admission during the period was enrolled. The source dataset documented exclusion of known systemic sclerosis, other connective-tissue disease, and Raynaud phenomenon, but it did not contain results from a dedicated protocol-mandated rheumatologic serology screening panel. Accordingly, occult or previously undiagnosed connective-tissue disease cannot be completely excluded.

The underlying data were acquired during routine hospitalization within a broader institutional clinical-research program conducted in the same department (“Clinical and biological predictors of heart-failure decompensation in hospitalized patients”), in which participants underwent standardized extended vascular phenotyping—including carotid ultrasonography—alongside usual clinical care. No study-directed therapeutic intervention was assigned. Every participant provided written informed consent at hospital admission, including authorization for the use of pseudonymized clinical, laboratory, vascular, imaging, and cognitive data for research and publication purposes. The Ethics Council of the Railway Clinical Hospital Timișoara approved the present retrospective secondary analysis and its publication on 30 July 2026 (approval no. 5967). The approval document explicitly covers research activity and data from the Internal Medicine II Department for the period from November 2024 through July 2026; therefore, the complete data-acquisition interval analyzed in this manuscript (1 July 2025–30 June 2026) falls within the period specified by the approval.

### 2.2. Clinical Variables and Blood-Pressure Assessment

Clinical variables included age, sex, years of education, body mass index (BMI), duration of hypertension, systolic blood pressure (SBP), diastolic blood pressure (DBP), mean arterial pressure, pulse pressure, number and classes of antihypertensive agents, statin use, active and former smoking, diabetes mellitus, dyslipidemia, chronic kidney disease, and established cardiovascular disease. All 217 patients were receiving at least one antihypertensive drug class in the database (range, 1–4 classes). Diabetes mellitus was available as a binary clinical variable. Standardized diabetes duration, retinopathy, peripheral neuropathy, and adjudicated diabetic kidney disease were not retained as variables in the analytic dataset; UACR and eGFR therefore could not be attributed specifically to diabetic microangiopathy. Consequently, subgroup analyses by diabetes duration or complication status could not be reconstructed reliably.

For the present analysis, the standardized in-hospital blood-pressure phenotype was classified as controlled when SBP was <140 mmHg and DBP was <90 mmHg and as uncontrolled when SBP was ≥140 mmHg and/or DBP was ≥90 mmHg. This threshold reproduces the prespecified classification stored in the clinical database. Throughout the manuscript, ‘controlled’ and ‘uncontrolled’ refer specifically to this standardized in-hospital blood-pressure phenotype and should not be interpreted as characterization of long-term or ambulatory blood-pressure control or as implying that 140/90 mmHg represents the optimal treatment target for every patient [1,2].

Blood pressure was measured on the morning of the vascular assessment, before the administration of antihypertensive medication, using a validated automated oscillometric device (Omron HEM-907XL, Omron Healthcare, Kyoto, Japan). Measurements were obtained with the patient seated, back supported and feet flat on the floor, after a rest period of at least 5 min in a quiet room, with the arm supported at heart level and a cuff size selected according to mid-arm circumference. Three readings were obtained at 1–2 min intervals, and the mean of the last two readings was used for analysis. Because all readings were obtained during hospitalization, they are referred to throughout as standardized in-hospital blood pressure and are not interchangeable with office measurements obtained in ambulatory practice or with ambulatory blood-pressure monitoring. Thus, the BP measurement session was temporally linked to the morning vascular-phenotyping assessment. The analytic dataset did not retain a standardized day-from-admission variable; therefore, the exact interval from admission to phenotyping and whether every participant was assessed on the same hospitalization day cannot be established retrospectively.

### 2.3. Laboratory Assessment

Venous blood samples were collected during hospitalization after an overnight fast of at least 8 h. Routine laboratory parameters were processed in the hospital’s central laboratory using certified assays on a Roche Cobas 8000 modular analyzer series (Roche Diagnostics, Mannheim, Germany). During the study period, glycated hemoglobin (HbA1c) testing could not be performed in the hospital laboratory because the required reagents were temporarily unavailable. Blood samples for HbA1c measurement were therefore collected during hospitalization and transferred to Bioclinica, a private laboratory in Timișoara, Romania, for analysis; the cost of the determination was borne by the patients. Laboratory variables analyzed included low-density lipoprotein cholesterol (LDL-C), HbA1c, estimated glomerular filtration rate (eGFR), urinary albumin-to-creatinine ratio (UACR) from a first-morning spot urine sample, and high-sensitivity C-reactive protein (hs-CRP). HbA1c was used for metabolic characterization and was not included in either primary multivariable association model or in the expanded cIMT sensitivity model; consequently, the principal adjusted association estimates do not depend on HbA1c values. Nevertheless, because the available analytic database comprised patients who completed the entire assessment bundle, access or willingness to complete externally processed testing could have influenced representation in the fully phenotyped cohort, and this is acknowledged as a potential selection mechanism. eGFR was calculated using the 2009 CKD-EPI creatinine equation [20]. UACR was analyzed continuously and summarized using the median and interquartile range because of its markedly skewed distribution. Completion of the full assessment bundle meant that all 217 participants included in the analytic cohort had an HbA1c result available. However, the source database did not retain the total number of hypertensive admissions during the study interval, the number who started extended phenotyping, the number who did not complete the bundle, or standardized reasons for noncompletion. These recruitment-flow counts therefore cannot be reconstructed retrospectively and are not imputed or estimated.

### 2.4. Nailfold Capillaroscopy and Composite Microvascular Score

Nailfold capillaroscopy was performed to characterize peripheral microvascular structure and flow, following the methodological principles endorsed by the international capillaroscopy literature regarding acclimatization, standardized image acquisition, operator training, and reproducible definitions of morphological abnormalities [21,22]. Examinations were carried out in a temperature-controlled room at 22–24 °C after an acclimatization period of at least 15 min. Participants were asked to avoid caffeine and nicotine on the morning of the examination, and the absence of nail cosmetics and of recent finger trauma was verified before acquisition. Images were acquired at ×200 magnification using immersion oil to improve optical resolution with a dedicated video capillaroscope (Videocap 3.0, DS Medica, Milan, Italy). The second to fifth fingers of both hands were examined, with two adjacent fields of 1 mm assessed per finger.

Quantitative parameters comprised capillary density expressed as capillaries per millimeter and averaged across all evaluable fields, erythrocyte velocity in millimeters per second, apical loop width in micrometers, the percentage of tortuous capillaries, and the percentage of dilated loops. Capillary dilation was defined as an apical loop diameter exceeding 20 µm and giant capillaries as loops exceeding 50 µm. Qualitative parameters comprised microhemorrhages, avascular areas defined as the absence of capillary loops over a distance of at least 500 µm, and ramified capillaries. Fields of inadequate optical quality were discarded before scoring. All images were read by trained observers blinded to the participants’ clinical characteristics, laboratory results, and carotid ultrasound findings. Formal duplicate blinded re-reading for a prespecified reproducibility sample was not embedded in this cohort; therefore, local intra- or inter-observer intraclass correlation coefficients, coefficients of variation, standard errors of measurement, and limits of agreement are unavailable.

A composite microvascular score ranging from 0 to 10 was derived for every participant by integrating capillary rarefaction, impaired erythrocyte flow, tortuosity, dilation, microhemorrhages, and avascular areas, with higher values indicating a greater abnormality burden. The component definitions and point allocation are provided in full in Table S1. Score construction and component cut-points were specified before outcome modeling. Distribution-informed cut-points were used only to create ordinal severity bands; standardized in-hospital BP phenotype, cIMT, receiver-operating-characteristic optimization, *p* values, and outcome-dependent weighting were not used to select thresholds or weights. Continuous components contributed 0–2 points and binary abnormalities 0–1 point, and no diagnostic score cut-off was defined. Because this locally constructed composite has not been externally validated, it was treated as an exploratory research index. Individual capillaroscopic variables were therefore retained in the descriptive and correlation analyses rather than being replaced by the composite alone.

### 2.5. Carotid Ultrasonography

Carotid ultrasonography was performed by experienced operators blinded to the capillaroscopic and cognitive findings, using a high-resolution ultrasound system (Philips EPIQ 7, Philips Healthcare, Best, The Netherlands) equipped with a 12 MHz linear-array transducer (L12-3). Examinations were carried out with the participant supine, and the neck slightly extended and rotated 45° away from the side examined, after at least 10 min of rest. Intima-media thickness was measured on the far wall of the distal 1 cm of both common carotid arteries, proximal to the bulb dilation, in an end-diastolic frame identified by simultaneous electrocardiographic gating. Three measurements were obtained on each side, and mean carotid intima–media thickness (cIMT) was calculated as the average of the right and left values.

Mean cIMT constituted the primary structural vascular outcome and was analyzed primarily as a continuous variable in order to preserve information and reduce dependence on a single threshold, an approach consistent with the recognized influence of age, sex, segment selection, and acquisition methodology on absolute cIMT values [14,15,16,17,18]. A value of cIMT ≥ 0.90 mm was additionally examined as an exploratory categorical marker of increased carotid wall thickness. The historical context for this cut-point is prior European hypertension guidance: the 2018 ESC/ESH guideline described carotid IMT > 0.9 mm as abnormal in the assessment of hypertension-mediated organ damage [4]. We used ≥0.90 mm only as a pragmatic exploratory operationalization of that historical convention, not as a contemporary universal diagnostic threshold. It was not equated with carotid plaque, since the Mannheim consensus explicitly distinguishes diffuse intima–media thickening from focal plaque, and contemporary risk assessment accords greater prognostic weight to plaque than to an isolated cIMT cut-off [15].

### 2.6. Endothelial Function and Arterial Stiffness

Endothelial and vascular function were characterized using brachial artery flow-mediated dilation (FMD), the reactive hyperemia index (RHI), and carotid–femoral pulse wave velocity (PWV). All three measurements were obtained in the morning, in a quiet temperature-controlled room, after an overnight fast of at least 8 h, abstention from caffeine and tobacco for at least 12 h, and at least 10 min of supine rest.

FMD was assessed according to published methodological standards [23,24] using the same Philips EPIQ 7 ultrasound system with the L12-3 linear transducer. The brachial artery was imaged longitudinally 2–5 cm above the antecubital fossa; after acquisition of the baseline diameter, an occlusion cuff placed on the forearm distal to the imaged segment was inflated to at least 50 mmHg above systolic pressure for 5 min, and the post-deflation diameter was recorded continuously for 3 min. FMD was expressed as the percentage change from baseline diameter at peak dilation.

RHI was measured by peripheral arterial tonometry using the EndoPAT 2000 device (Itamar Medical, Caesarea, Israel), with the post-occlusion to pre-occlusion pulse amplitude ratio computed automatically and normalized to the contralateral control arm. Carotid–femoral PWV was measured by a validated tonometric method using the SphygmoCor XCEL system (AtCor Medical, Sydney, Australia), with transit distance estimated as 80% of the direct carotid–femoral surface distance, and was interpreted as a continuous index of aortic stiffness in line with established expert recommendations [25,26]. For each modality, replicate measurements were obtained and averaged. All operators performing vascular function testing were blinded to the capillaroscopic and cognitive data.

### 2.7. Cognitive Assessment

Cognitive performance was assessed using the Romanian version of the Montreal Cognitive Assessment (MoCA, version 7.1), administered by trained personnel and reported on the conventional 0–30 scale [27]. One point was added for participants with 12 or fewer years of formal education, in accordance with the standard scoring instructions. MoCA was analyzed continuously for the principal association analyses in order to avoid loss of information; for descriptive purposes, cognitive impairment was defined as a total score below 26 points.

### 2.8. Outcomes

The principal clinical outcome was the uncontrolled standardized in-hospital blood-pressure phenotype defined in Section 2.2. The principal structural vascular outcome was the mean cIMT analyzed as a continuous variable. A cIMT value ≥ 0.90 mm was a secondary, exploratory outcome.

### 2.9. Sample Size

No a priori sample-size calculation was performed because this study was a secondary analysis of the fully phenotyped records available in the database for the 12-month data-acquisition interval. Accordingly, the sample size was determined by data availability rather than by a prospectively calculated target. For the primary continuous outcome, 217 participants and seven covariates yield approximately 31 observations per covariate, supporting stable multivariable linear estimation. For the exploratory binary cIMT endpoint, only 38 participants met the ≥0.90 mm threshold, corresponding to fewer than eight events per variable; the model for this endpoint was therefore deliberately restricted to five covariates, and its results are presented as hypothesis-generating rather than confirmatory.

### 2.10. Statistical Analysis

Continuous variables were summarized as mean ± standard deviation when approximately symmetric and as median [interquartile range] when markedly skewed or ordinal; distributional assumptions were examined graphically and with the Shapiro–Wilk test. Categorical variables were summarized as *n* (%). Between-group comparisons used Welch *t*-tests for approximately symmetric continuous variables, Mann–Whitney U tests for skewed or ordinal variables, and chi-square or Fisher exact tests for categorical variables, as appropriate. Spearman rank correlation coefficients quantified monotonic associations between cIMT and vascular, renal, inflammatory, metabolic, and cognitive variables, and the Benjamini–Hochberg procedure was applied to control the false-discovery rate (FDR) across the cIMT correlation matrix [28].

A multivariable logistic regression model evaluated the association between the microvascular score and the uncontrolled standardized in-hospital BP phenotype. Because SBP and DBP mathematically define this outcome, they were not entered as predictors. Covariates were selected a priori on clinical grounds and comprised age, sex, BMI, hypertension duration, diabetes, active smoking, eGFR, and microvascular score. Effects are reported as adjusted odds ratios (ORs) with 95% confidence intervals (CIs). This model was specified to estimate an adjusted association rather than to develop or validate a diagnostic prediction rule; no score threshold, classification algorithm, or diagnostic-performance claim was derived from it.

For the primary structural analysis, robust linear regression with heteroscedasticity-consistent (HC3) standard errors modeled mean cIMT as a continuous outcome. The primary continuous-cIMT model included age, sex, hypertension duration, diabetes, LDL-C, eGFR, and microvascular score. Variance inflation factors were examined to assess collinearity, with values above 5 considered indicative of problematic multicollinearity. An expanded sensitivity model additionally included BMI, contemporaneous SBP, and active smoking to test the dependence of the microvascular-score estimate on contemporaneous pressure exposure and conventional risk factors. The primary model was defined a priori as a core confounder-adjusted model capturing demographic factors, cumulative hypertension burden, diabetes, lipid exposure, and renal function. BMI, contemporaneous SBP, and active smoking were added together in the expanded model as a deliberately more conservative robustness analysis of additional conventional and current-exposure confounding. This hierarchy is analytic rather than causal: interpretation is based on both models, and attenuation in the expanded model is treated as evidence of model sensitivity rather than as proof of an independent cIMT relationship. A parsimonious logistic model comprising age, sex, hypertension duration, diabetes, and microvascular score was used for the exploratory cIMT ≥ 0.90 mm endpoint because of the limited number of events. Effect modification by sex and by diabetes was explored using multiplicative interaction terms. No automated stepwise variable-selection procedure, outcome-driven covariate selection, or optimization of the microvascular score was used at any stage.

All tests were two-sided, and *p* < 0.05 was considered statistically significant, with FDR-adjusted *p* values used for the designated cIMT correlation matrix. The 217-record analytic dataset contained no missing values for the variables reported, and imputation was therefore not required. Statistical inference focused on effect estimates, 95% CIs, consistency across vascular domains, and sensitivity to model specification rather than on isolated *p* values. Statistical analyses were performed with Python 3.13.5 using NumPy 2.3.5, SciPy 1.17.0, and statsmodels 0.14.6.

### 2.11. Ethical Considerations

All participants provided written informed consent at hospital admission. Consent covered the use of pseudonymized clinical, laboratory, vascular, imaging, and cognitive data for research and publication purposes, and participants were informed that refusal would not affect the standard of care received.

The present retrospective secondary analysis and its publication were approved by the Ethics Council of the Railway Clinical Hospital Timișoara, Romania, on 30 July 2026 (approval no. 5967). The approval document expressly covers research activity and use of data from the Internal Medicine II Department for the period from November 2024 through July 2026. The acquisition window of the present cohort (1 July 2025–30 June 2026) is therefore entirely contained within the period specified by the Ethics Council. The approval date represents the date of authorization for the present retrospective secondary analysis and publication of already collected, consented data within that covered period and is not the start date of patient data acquisition. The study was conducted in accordance with the ethical principles of the Declaration of Helsinki and applicable institutional and national data-protection legislation. All patient records were pseudonymized before analysis, and no directly identifying information was accessible to the authors performing the statistical analysis.

## 3. Results

### 3.1. Study Population

The analytic cohort included 217 fully phenotyped treated adults with hypertension. Mean age was 61.3 ± 9.9 years, 110 (50.7%) were men, mean BMI was 29.3 ± 4.0 kg/m^2^, and mean hypertension duration was 10.8 ± 6.7 years. Mean standardized in-hospital BP was 142.6 ± 13.3/82.5 ± 7.9 mmHg. Based on the operational definition specified in Section 2.2, 145 patients (66.8%) had an uncontrolled standardized in-hospital BP phenotype and 72 (33.2%) had a controlled standardized in-hospital BP phenotype. Every patient received at least one antihypertensive class; the median number was 2 [IQR 2–3]. Because the source database was not a screening registry, a denominator of all potentially eligible hypertensive admissions was unavailable and no participant flow diagram is presented.

Diabetes mellitus was present in 53 (24.4%), dyslipidemia in 129 (59.4%), chronic kidney disease in 31 (14.3%), and established cardiovascular disease in 41 (18.9%). Mean cIMT was 0.776 ± 0.112 mm; 38 patients (17.5%) had cIMT ≥ 0.90 mm. The median composite microvascular score was 3 [1–4], with an observed range of 0 to 8 points. Baseline characteristics are summarized in Table 1.

Among the 53 participants with diabetes, a descriptive stratification by diabetes duration, retinopathy, neuropathy, or adjudicated diabetic kidney disease could not be performed because these variables were not standardized in the source analytic dataset. Diabetes status was therefore handled as a prespecified covariate in the multivariable models and as an exploratory effect modifier (Section 3.5; Table S5), rather than by post hoc reconstruction of unverified complication categories.

### 3.2. Integrated Vascular Phenotype According to the Standardized In-Hospital Blood-Pressure Phenotype

Patients with an uncontrolled standardized in-hospital BP phenotype were older than those with a controlled phenotype (62.87 ± 10.06 vs. 58.03 ± 8.73 years; *p* < 0.001), whereas BMI and hypertension duration did not differ significantly. A coherent adverse vascular pattern was present in the uncontrolled group: lower FMD and RHI, higher PWV, lower capillary density and erythrocyte velocity, greater tortuosity and loop dilation, higher UACR, lower MoCA score, and greater cIMT (Table 2). High-sensitivity C-reactive protein did not differ significantly between the two groups.

The median microvascular score was 3 [2–4] in the uncontrolled phenotype versus 2 [1–3] in the controlled phenotype (*p* < 0.001). Avascular areas were also more frequent (17.9% vs. 5.6%; *p* = 0.013). Mean cIMT was 0.79 ± 0.12 mm in the uncontrolled phenotype and 0.74 ± 0.10 mm in the controlled phenotype (*p* = 0.001).

### 3.3. Microvascular Abnormalities and Carotid Wall Thickness

**Figure 1:**
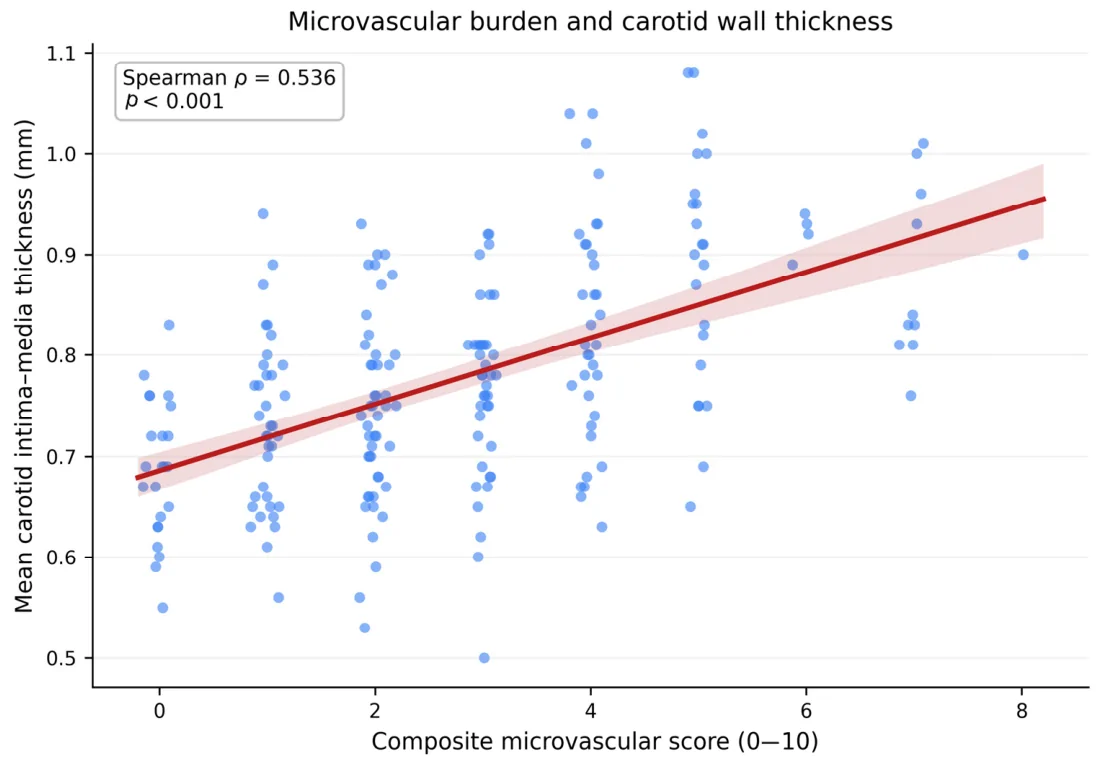
Relationship between composite nailfold microvascular abnormality burden and mean carotid intima–media thickness. Points represent individual participants; the fitted line is shown for visualization. The inferential association is summarized using Spearman rank correlation (ρ = 0.536, *p* < 0.001).

The composite microvascular score showed a moderate-to-strong positive monotonic association with mean cIMT (Spearman ρ = 0.536; raw *p* < 0.001; FDR-adjusted *p* < 0.001; Figure 1). The direction of associations across individual capillaroscopic measures was internally consistent: greater cIMT was associated with lower capillary density (ρ = −0.384), lower erythrocyte velocity (ρ = −0.455), greater apical width (ρ = 0.211), greater tortuosity (ρ = 0.383), and a higher percentage of dilated loops (ρ = 0.363). Each association remained significant after FDR correction. Descriptively, the absolute score–cIMT correlation was greater than that of capillary density alone (|ρ| = 0.536 vs. 0.384). Because capillary density is itself a component of the composite, this contrast is supportive only and does not establish incremental predictive or diagnostic value.

**Figure 2:**
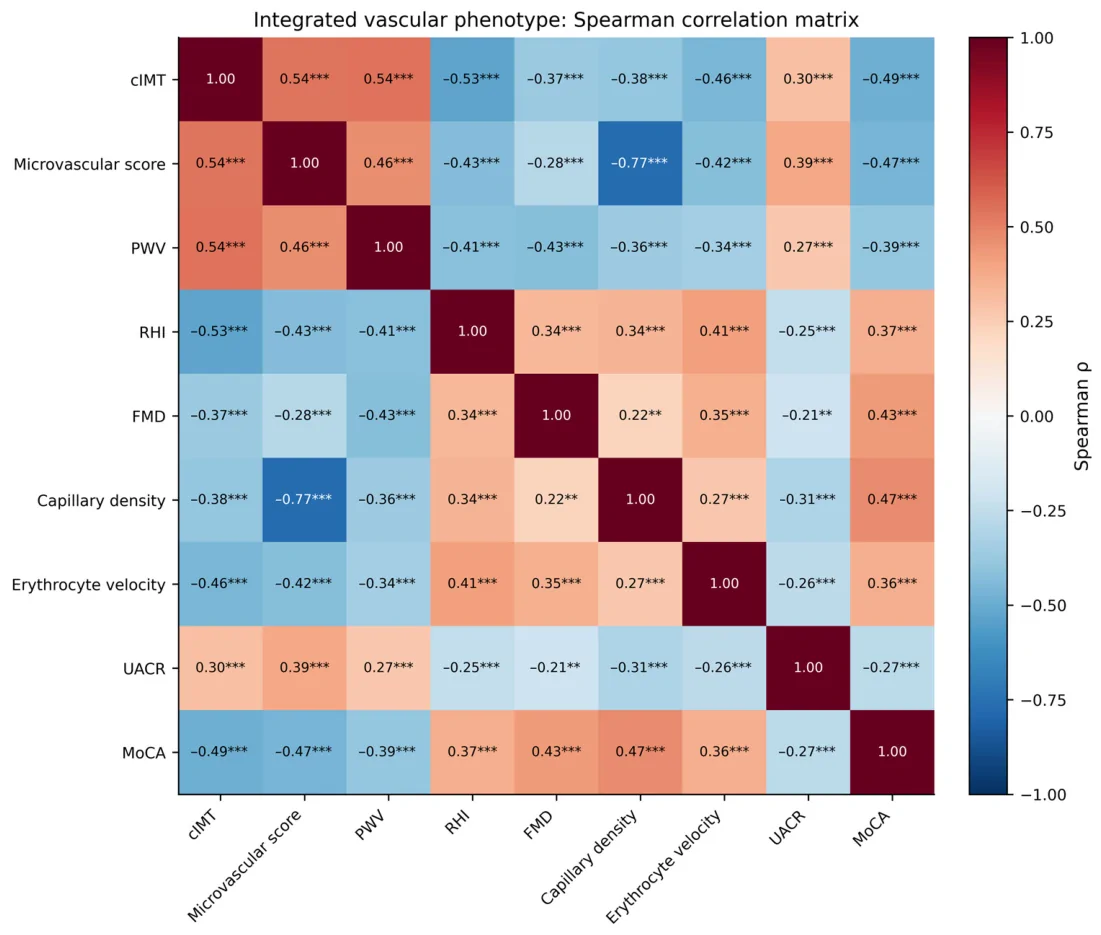
Integrated vascular phenotype: Spearman correlation matrix. Values are Spearman rho coefficients. Asterisks denote statistical significance based on the underlying two-sided tests (** *p* < 0.01; *** *p* < 0.001). cIMT, carotid intima–media thickness; FMD, flow-mediated dilation; MoCA, Montreal Cognitive Assessment; PWV, pulse wave velocity; RHI, reactive hyperemia index; UACR, urinary albumin-to-creatinine ratio.

cIMT was also associated with other vascular and end-organ markers: PWV (ρ = 0.540), RHI (ρ = −0.531), FMD (ρ = −0.373), eGFR (ρ = −0.546), UACR (ρ = 0.302), and MoCA (ρ = −0.489), all with FDR-adjusted *p* < 0.001. Age showed the strongest correlation with cIMT (ρ = 0.686), while SBP (ρ = 0.315) and LDL-C (ρ = 0.197) showed weaker but significant positive associations. Figure 2 depicts the principal vascular correlation matrix; the complete cIMT correlation analysis is provided in Supplementary Table S2.

### 3.4. Exploratory Phenotype Associated with cIMT ≥ 0.90 mm

Thirty-eight patients (17.5%) had cIMT ≥ 0.90 mm. Compared with participants below this threshold, they were older (71.89 ± 6.71 vs. 59.01 ± 8.94 years; *p* < 0.001), had longer hypertension duration (14.20 ± 6.67 vs. 10.05 ± 6.53 years; *p* < 0.001), and showed a markedly adverse multidomain vascular phenotype. FMD and RHI were lower, PWV was higher, capillary density and erythrocyte velocity were lower, and tortuosity, loop dilation, UACR, and microvascular score were higher (Table 3).

**Figure 3:**
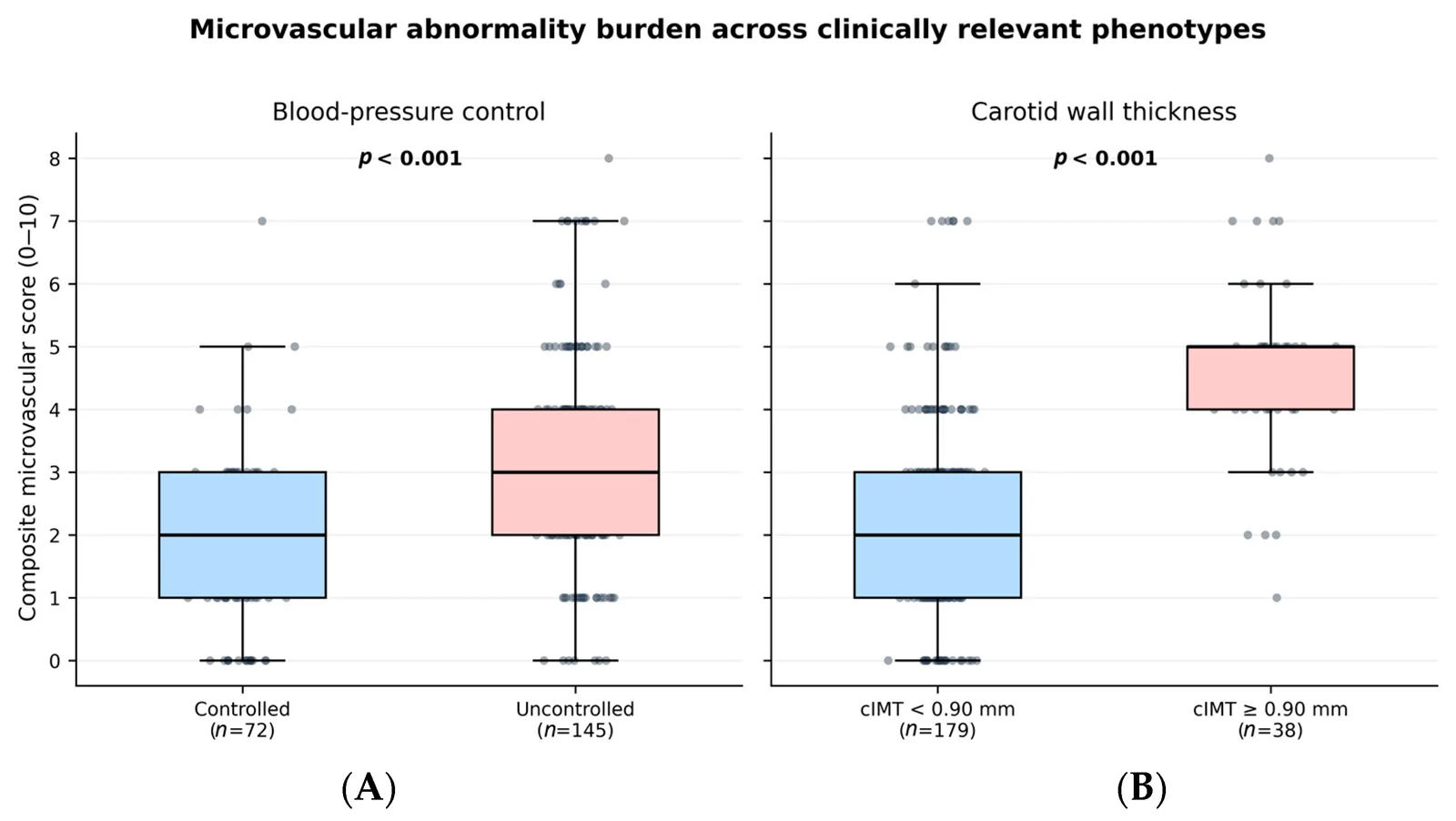
Distribution of composite microvascular score across clinically relevant phenotypes. (**A**) Controlled versus uncontrolled standardized in-hospital BP phenotype. (**B**) cIMT < 0.90 versus ≥0.90 mm. Boxplots show median and interquartile range with individual observations overlaid; *p* values derive from Mann–Whitney U tests. Blue indicates the controlled/lower-cIMT groups, pink indicates the uncontrolled/higher-cIMT groups, and gray dots represent individual participants.

The median microvascular score was 5 [4–5] in patients with cIMT ≥ 0.90 mm and 2 [1–3] among those with lower cIMT (*p* < 0.001). Figure 3 summarizes the score distributions by standardized in-hospital BP phenotype (Figure 3A) and the exploratory cIMT threshold (Figure 3B). Avascular areas were present in 36.8% versus 8.9%, respectively (*p* < 0.001). Cognitive impairment was also more frequent in the higher-cIMT group (73.7% vs. 27.9%; *p* < 0.001). These findings are descriptive and do not establish that cIMT itself mediates renal or cognitive abnormalities.

### 3.5. Multivariable Analyses

**Figure 4:**
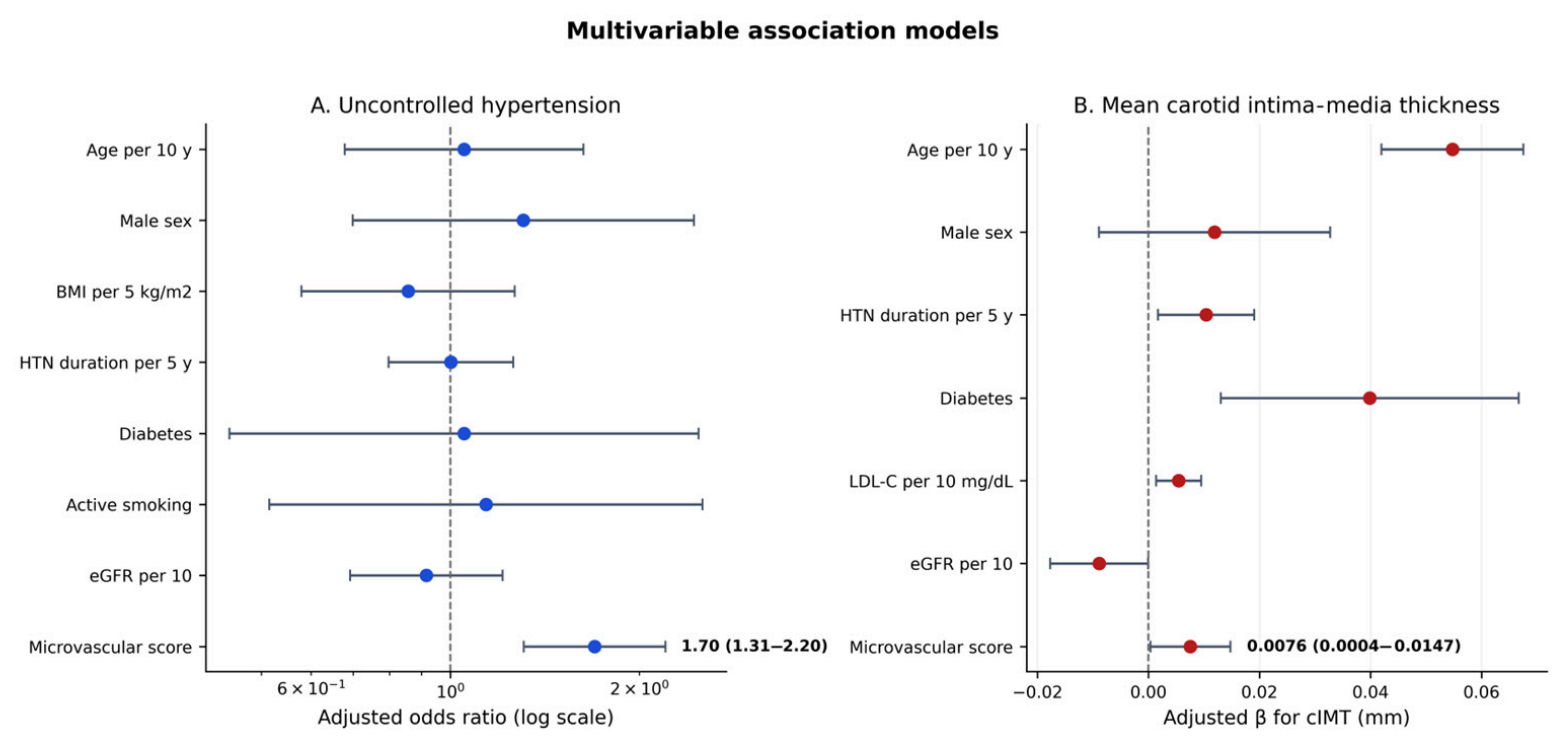
Multivariable association models. (**A**) Adjusted odds ratios and 95% confidence intervals for the uncontrolled standardized in-hospital BP phenotype. (**B**) Adjusted regression coefficients and 95% confidence intervals for mean cIMT in the primary robust linear model. Blue markers indicate estimates from the logistic regression model for the uncontrolled BP phenotype, whereas red markers indicate estimates from the robust linear regression model for mean cIMT; horizontal lines represent 95% confidence intervals. The microvascular-score estimates are annotated. cIMT, carotid intima-media thickness; eGFR, estimated glomerular filtration rate; HTN, hypertension; LDL-C, low-density lipoprotein cholesterol.

In the multivariable logistic model for the uncontrolled standardized in-hospital BP phenotype, each one-point increase in the microvascular score was associated with 70% higher adjusted odds of the uncontrolled standardized in-hospital BP phenotype (adjusted OR 1.70, 95% CI 1.31–2.20; *p* < 0.001) after accounting for age, sex, BMI, hypertension duration, diabetes, active smoking, and eGFR. None of the remaining covariates reached statistical significance in that model (Table 4; Figure 4A).

In the primary robust linear model for continuous cIMT, the microvascular score remained associated with greater mean cIMT after adjustment for age, sex, hypertension duration, diabetes, LDL-C, and eGFR: adjusted β = 0.0076 mm per score point (95% CI 0.0004–0.0147; *p* = 0.040). Age, hypertension duration, diabetes, LDL-C, and eGFR also retained associations with cIMT in the adjusted model. The model explained 57.2% of the variance in cIMT (adjusted R^2^ = 55.8%), and all variance inflation factors were <2, arguing against problematic collinearity (Figure 4B).

The association between microvascular score and continuous cIMT was attenuated in the expanded sensitivity model that additionally included BMI, contemporaneous SBP, and active smoking (β = 0.0058 mm per point, 95% CI −0.0018 to 0.0134; *p* = 0.138). This attenuation indicates that part of the unadjusted microvascular–cIMT relationship may reflect shared variance with current pressure exposure and conventional risk factors rather than an entirely independent pathway. The total model R^2^ increased only from 0.572 to 0.580 (Table S3).

For the exploratory cIMT ≥ 0.90 mm endpoint, a parsimonious logistic model showed an adjusted association with microvascular score (OR 1.48 per point, 95% CI 1.07–2.04; *p* = 0.017), whereas age (OR 5.31 per 10 years, 95% CI 2.56–10.99; *p* < 0.001) and hypertension duration (OR 1.69 per 5 years, 95% CI 1.16–2.47; *p* = 0.007) were dominant determinants (Table S4). No statistically significant interaction was found between microvascular score and sex or diabetes for the uncontrolled standardized in-hospital BP phenotype; interaction tests for continuous cIMT were likewise non-significant, although the score-by-sex interaction approached but did not cross the conventional threshold (*p* = 0.057). These subgroup analyses should be regarded as exploratory because the study was not powered for interaction testing (Table S5).

## 4. Discussion

### 4.1. Principal Findings

This integrated vascular-phenotyping study produced four principal observations. First, treated patients with an uncontrolled standardized in-hospital BP phenotype had a distinctly adverse nailfold microvascular phenotype, with lower capillary density and erythrocyte velocity, greater tortuosity and loop dilation, more frequent avascular areas, and a higher composite abnormality score. Second, the composite microvascular score correlated moderately to strongly with mean cIMT and remained associated with cIMT in a clinically adjusted continuous model. Third, the same patients showed concordant abnormalities in FMD, RHI, PWV, albuminuria, renal function, and cognitive performance, supporting the presence of a multidomain systemic vascular phenotype. Fourth, the cIMT association was attenuated when contemporaneous SBP, BMI, and smoking were added to an expanded sensitivity model. This attenuation indicates that part of the relationship between nailfold abnormalities and carotid wall thickness is shared with conventional cardiovascular risk factors and contemporaneous pressure exposure.

The strongest adjusted signal was the association between microvascular abnormality burden and the uncontrolled standardized in-hospital BP phenotype. The adjusted odds of the uncontrolled standardized in-hospital BP phenotype increased by approximately 70% per one-point increase in the 0–10 score, despite adjustment for demographic factors, obesity, hypertension duration, diabetes, smoking, and renal function. Because the outcome is cross-sectional and based on in-hospital measurements, this result should not be interpreted as a predictive effect. It does, however, show that the microvascular score retained an adjusted association after accounting for the routine clinical variables included in the model.

### 4.2. Microvascular Rarefaction and Morphological Remodeling in Hypertension

The capillary-density findings are consistent with the classical concept of microvascular rarefaction in hypertension. Noon et al. reported impaired dermal vasodilatation and fewer capillaries in young adults expressing a familial predisposition to high blood pressure [5]. Antonios and colleagues subsequently demonstrated capillary rarefaction in borderline and established essential hypertension, including evidence for a structural component that could not be explained solely by transient non-perfusion [6,7]. Serné et al. further separated functional from structural rarefaction using recruitment maneuvers and concluded that both mechanisms contribute to impaired skin capillary recruitment [8]. These studies are mechanistically important because fewer parallel microvascular channels increase effective resistance and may amplify the hemodynamic load transmitted to the microcirculation.

Treatment does not necessarily normalize this phenotype. Penna et al. observed persistent rarefaction in treated essential hypertension [9], and subsequent reviews have emphasized that microvascular density can reflect long-term remodeling rather than only instantaneous vasomotor tone [10]. Our patients were all treated with at least one antihypertensive class, yet capillary density remained significantly lower and the composite score substantially higher among those with the uncontrolled standardized in-hospital BP phenotype. The result is therefore compatible with a model in which higher contemporaneous in-hospital pressure, cumulative pre-treatment exposure, vascular aging, and potentially irreversible structural remodeling contribute simultaneously.

The observed abnormalities extended beyond density. Lower erythrocyte velocity, increased tortuosity, loop dilation, and avascular areas suggest a broader perturbation of capillary architecture and perfusion. Mishra et al. reported more capillaroscopic abnormalities in newly diagnosed hypertensive patients and relationships with microvascular changes in other organs [11]. A systematic review of non-rheumatic conditions similarly concluded that reduced density and non-specific morphological abnormalities are encountered outside systemic sclerosis, but it cautioned that comorbidities can influence their interpretation [12]. This is why our composite score should be viewed as a research index rather than a disease-specific capillaroscopic pattern.

The cardiovascular capillaroscopy literature remains heterogeneous with respect to devices, magnification, acclimatization, finger selection, image analysis, and abnormality definitions [13]. Rigorous and fully specified acquisition reporting is therefore essential, and the acquisition parameters used here are described in Section 2.4 in sufficient detail to allow direct replication. External reproducibility of the composite score will be particularly important if the signal is to move beyond a single-center hypothesis-generating observation.

### 4.3. Why Might the Microvascular Phenotype Track the Standardized In-Hospital Blood-Pressure Phenotype?

Several non-mutually exclusive mechanisms can explain the association with the uncontrolled standardized in-hospital BP phenotype. Chronic pressure load promotes endothelial dysfunction, oxidative stress, inflammation, altered nitric-oxide signaling, vascular smooth-muscle hypertrophy, inward remodeling of resistance vessels, and impaired angiogenic balance. Structural rarefaction can then increase peripheral resistance, creating a potential feed-forward loop in which hypertension damages the microcirculation and a reduced microvascular network helps sustain elevated resistance. The cross-sectional design cannot resolve the direction of this relationship, and bidirectionality is biologically more plausible than a single causal sequence.

A second explanation is that capillaroscopy may integrate time. Single SBP and DBP readings are highly variable snapshots, whereas structural abnormalities such as rarefaction or persistent tortuosity may encode longer cumulative exposure. This distinction is clinically relevant because a single in-hospital or clinic BP session does not fully characterize 24 h BP load, nocturnal hypertension, morning surge, or long-term adherence. Conversely, a single high measurement may reflect transient stress or white-coat physiology. Future studies should pair quantitative capillaroscopy with ambulatory blood-pressure monitoring (ABPM), home measurements, medication adherence, and longitudinal BP trajectories.

The absence of statistically significant adjusted associations for age, BMI, diabetes, smoking, or eGFR in the uncontrolled standardized in-hospital BP logistic model should not be interpreted as evidence that these factors are unimportant. The model addresses conditional associations within this specific cohort, and its purpose was to determine whether the microvascular score retained a signal after adjustment. A sample of 217 participants also limits precision for smaller effects and interaction testing.

### 4.4. A Microvascular-Macrovascular Continuum: cIMT, Endothelial Function, and Arterial Stiffness

The second major finding was concordance between the nailfold phenotype and carotid wall morphology. The microvascular score correlated with cIMT at ρ = 0.536, while lower capillary density and erythrocyte velocity correlated inversely with cIMT. This pattern is consistent with the concept that micro- and macrovascular changes are interconnected manifestations of cumulative vascular stress rather than isolated phenomena.

Endothelial-function measures reinforced this interpretation. Greater cIMT was associated with lower FMD and lower RHI. FMD is a widely used measure of conduit-artery vasodilator responsiveness, and standardized methodology is critical because cuff position, shear stimulus, baseline diameter, and analytic technique influence the result [23,24]. Across broader cardiovascular populations, lower FMD has been associated with future cardiovascular risk, although its biological interpretation is not reducible to nitric oxide alone [29,30]. Digital reactive-hyperemia measures are likewise related to cardiovascular risk-factor burden and have shown prognostic associations in selected high-risk populations [31,32,33]. Thus, the direction of our FMD and RHI findings is physiologically coherent with the adverse capillaroscopic phenotype.

PWV displayed one of the strongest correlations with cIMT (ρ = 0.540). Carotid–femoral PWV is the reference non-invasive measure of aortic stiffness [25,26], and increased stiffness predicts cardiovascular events and mortality [34]. Longitudinal data also support a bidirectional relationship between arterial stiffness and pressure progression: aortic stiffness can precede incident hypertension, while persistent hypertension accelerates stiffening [35,36]. In the Framingham Heart Study, aortic stiffness was independently associated with incident cardiovascular events [37], and microvascular function contributed to that relationship [38]. Our concurrent findings of higher PWV, worse RHI and FMD, and greater capillaroscopic abnormality burden therefore fit an integrated vascular model.

Mechanistically, large-artery stiffening increases pulsatile energy transmission into low-resistance microvascular beds, while microvascular dysfunction can increase wave reflection and peripheral resistance. Endothelial dysfunction, inflammation, collagen deposition, elastin fragmentation, and vascular calcification may affect multiple vascular compartments in parallel. The present data cannot determine which component is upstream, but their cross-sectional concordance argues against interpreting any single vascular biomarker in isolation.

### 4.5. Interpreting cIMT in 2026: Signal, Thresholds, and Confounding

cIMT requires especially careful interpretation. Ultrasound measurement is technically standardized, but values vary by carotid segment, wall, age, sex, and measurement convention [14,15]. Epidemiologic meta-analysis has linked higher cIMT with future myocardial infarction and stroke [16], and contemporary synthesis confirms that different cIMT definitions have different strengths of association with future cardiovascular disease [18]. At the same time, focal carotid plaque represents a distinct atherosclerotic phenotype and is generally more directly actionable for risk reclassification than diffuse wall thickness. For this reason, we deliberately treated continuous mean cIMT as the primary structural outcome. The secondary ≥ 0.90 mm analysis was retained only for historical comparability with earlier European hypertension guidance, which regarded carotid IMT > 0.9 mm as abnormal in HMOD assessment [4]; it should not be interpreted as a current universal threshold or as equivalent to plaque.

The adjusted analyses materially improve the credibility of the cIMT findings. In the primary robust model, each one-point increase in microvascular score corresponded to approximately 0.0076 mm higher cIMT after adjustment for age, sex, hypertension duration, diabetes, LDL-C, and eGFR. The magnitude of this effect should be read carefully: across the observed score range of 0 to 8 points it amounts to roughly 0.06 mm, which is small relative to the between-participant spread in cIMT (SD 0.112 mm) and to the adjusted age effect of approximately 0.055 mm per decade. Most of the crude association therefore reflects variance shared with age and other established determinants, and the residual microvascular-score coefficient is modest. Age was, appropriately, the dominant covariate. Hypertension duration, diabetes, LDL-C, and lower eGFR also retained adjusted associations, indicating that the model captured expected biology rather than assigning an implausibly dominant role to capillaroscopy.

In the expanded sensitivity model additionally including BMI, contemporaneous SBP, and active smoking, the microvascular-score estimate was attenuated and no longer met the conventional threshold for statistical significance because contemporaneous SBP may capture part of the same cumulative hemodynamic burden reflected by both microvascular abnormalities and cIMT, whereas it may also act as an important confounding factor, the expanded model should be interpreted as a sensitivity analysis rather than as a definitive decomposition of causal pathways. The attenuation nevertheless indicates that the microvascular–cIMT association is modest and sensitive to inclusion of conventional cardiovascular risk factors and contemporaneous pressure exposure; it should not be interpreted as a fully independent relationship.

The exploratory threshold analysis gives the same qualitative message without replacing the continuous analysis. Patients with cIMT ≥ 0.90 mm had substantially higher microvascular scores, more avascular areas, poorer endothelial function, higher PWV, and more renal/cognitive abnormalities. In the parsimonious logistic model, the score remained associated with the threshold, but age and hypertension duration were much stronger determinants. This hierarchy is clinically plausible and argues against an overfitted claim that nailfold morphology supersedes established risk factors.

The association between hypertension and greater carotid wall thickness is supported by longitudinal evidence. Higher cIMT has been associated with first stroke among patients with hypertension [39], while ELSA-Brasil data have shown that cIMT itself can precede incident hypertension in initially non-hypertensive adults [40]. These reciprocal observations further support a shared vascular continuum rather than a simple one-way causal chain.

### 4.6. Renal Microvascular Injury and the Systemic Vascular Phenotype

Renal findings provide another layer of biological coherence. The uncontrolled standardized in-hospital BP phenotype was accompanied by higher UACR, and cIMT correlated positively with UACR and inversely with eGFR. Albuminuria is not merely a renal laboratory abnormality; it is widely interpreted as a marker of glomerular and systemic endothelial injury. In essential hypertension, microalbuminuria has historically been linked to other forms of target-organ damage [41], and large cardiovascular cohorts have shown a graded association between albuminuria and cardiovascular events even below traditional microalbuminuria thresholds [42].

The simultaneous presence of nailfold abnormalities, albuminuria, arterial stiffness, and greater carotid wall thickness is therefore consistent with a systemic microvascular and endothelial phenotype. hs-CRP did not differ by the standardized in-hospital BP phenotype and was only modestly higher in participants with cIMT ≥ 0.90 mm. This isolated hs-CRP finding should not be interpreted as evidence against an inflammatory contribution: IL-6, TNF-α, endothelial-activation biomarkers, and oxidative-stress markers were not measured, and these pathways may capture biology not reflected by hs-CRP alone. Diabetes was present in approximately one quarter of the cohort and can independently affect both the renal and nailfold microcirculation. Although diabetes status was included in the multivariable models and did not significantly modify the main associations in exploratory interaction testing, the dataset did not retain standardized diabetes duration or microvascular-complication variables. Residual confounding by cumulative glycemic exposure, retinopathy, neuropathy, diabetic kidney disease, and diabetes-specific medication exposure therefore remains possible.

### 4.7. Cognitive Findings and the Brain-Vascular Axis

The inverse association of cIMT and microvascular abnormality burden with MoCA score is clinically intriguing but should be interpreted cautiously. Hypertension is a recognized risk factor for vascular cognitive impairment and dementia through pathways that include cerebral small-vessel disease, microvascular rarefaction, endothelial dysfunction, blood–brain barrier disruption, ischemic injury, and neuroinflammation [43,44]. Intensive BP treatment has reduced the incidence of mild cognitive impairment in randomized evidence, reinforcing the clinical relevance of the BP–brain relationship [45].

Our study did not include brain MRI, formal neuropsychological batteries, adjudicated dementia diagnoses, or longitudinal cognitive outcomes. MoCA is a screening instrument [27], and the cross-sectional associations could reflect age, education, renal disease, prior vascular injury, or other unmeasured factors. The cognitive result is therefore best viewed as a convergent end-organ signal rather than evidence that nailfold or carotid abnormalities cause cognitive impairment. A future multimodal study combining ABPM, retinal or nailfold microvascular imaging, carotid plaque assessment, brain MRI markers of small-vessel disease, and serial cognition would be well positioned to test this hypothesis.

### 4.8. Potential Clinical Implications

The present results do not justify using nailfold capillaroscopy as a standalone diagnostic test for long-term or ambulatory BP control, carotid atherosclerosis, or HMOD. Current hypertension guidelines do not include nailfold capillaroscopy among routine HMOD tests [1,2]. The most appropriate interpretation is therefore complementary phenotyping: an abnormal peripheral microvascular pattern may identify a patient in whom the clinician should pay closer attention to the broader vascular context rather than assuming that a single clinic BP value fully represents vascular health. The observed association is specifically with the protocolized in-hospital BP phenotype; it does not establish an association with 24 h, home, or longitudinal BP control and cannot distinguish sustained uncontrolled hypertension from white-coat or masked uncontrolled BP patterns.

Capillaroscopy has practical attractions. It is non-invasive, repeatable, radiation-free, and allows direct visualization of living microvessels. In settings where the technique is already available, standardized quantitative acquisition could potentially enrich research phenotyping at low incremental burden. However, clinical translation requires evidence that capillaroscopic metrics are reproducible, add discrimination or reclassification beyond standard risk factors, respond meaningfully to intervention, and predict hard cardiovascular or renal outcomes. Those criteria have not yet been satisfied.

The composite score warrants particular restraint. Composite indices can increase signal-to-noise ratio when multiple abnormalities represent the same latent construct, but they can also conceal the behavior of individual components and overfit a single dataset. The strong inverse correlation between the score and capillary density (Figure 2; Table S2) indicates that rarefaction contributes materially to the composite, while the composite also incorporates other morphological elements. Although the absolute correlation with cIMT was numerically larger for the composite score than for capillary density alone (|ρ| = 0.536 vs. 0.384), this within-dataset descriptive contrast cannot establish incremental value because capillary density is included in the score. External validation should predefine component weights and test calibration, reproducibility, and incremental value rather than re-deriving an optimal score in every cohort. A proper external-validation program should lock the present score definition, component thresholds, and weights before analysis; apply them unchanged in an independent consecutively recruited cohort; quantify construct validity against individual capillaroscopic measures and other vascular domains; and evaluate whether the score adds information beyond conventional risk factors without re-deriving an “optimal” score in the validation sample.

### 4.9. Strengths

The study has several strengths. First, all 217 participants had complete data across clinical, biochemical, microvascular, endothelial, arterial-stiffness, carotid, renal, and cognitive domains, eliminating missing-data imputation as a source of uncertainty within the analytic cohort. Second, cIMT was modeled continuously as the principal structural outcome, reducing dependence on arbitrary categorization. Third, the analysis incorporated FDR correction for the correlation matrix, clinically specified multivariable adjustment, robust HC3 standard errors, collinearity assessment, an expanded sensitivity model, and exploratory interaction testing rather than relying solely on unadjusted *p* values. Fourth, the composite score was constructed without outcome-based optimization and was accompanied by reporting of its individual capillaroscopic components. Fifth, the direction of the findings was internally coherent across multiple vascular beds. Finally, explicit reporting of the attenuated estimate in the expanded sensitivity model supports a cautious interpretation of the cIMT association.

### 4.10. Limitations

Several limitations are important. First, the cross-sectional design precludes temporal or causal inference. The study cannot determine whether microvascular abnormalities contribute to the observed standardized in-hospital BP phenotype, result from cumulative pressure exposure, or both.

Second, this was a single-center, fully phenotyped cohort of patients hospitalized in an internal medicine department. The frequency of the uncontrolled standardized in-hospital BP phenotype and multimorbidity is therefore likely to differ from community-based or primary-care populations, limiting external generalizability. The source database was not designed as a screening registry and did not retain the total number of hypertensive admissions assessed for eligibility or detailed reasons for noncompletion of extended phenotyping. A formal participant flow diagram therefore cannot be reconstructed, the sample should not be interpreted as consecutive, and residual selection bias cannot be excluded. In addition, the principal reason for hospitalization was not systematically coded in the analytic dataset, so the admission-diagnosis spectrum cannot be quantified reliably.

Third, the standardized in-hospital BP phenotype was classified using a single protocolized morning measurement session rather than ambulatory blood-pressure monitoring or systematic home monitoring. Although the protocol used repeated measurements under controlled conditions, it does not characterize 24 h pressure load. White-coat and masked BP patterns, nocturnal hypertension, and blood-pressure variability could not be characterized. Medication adherence, dose intensity, timing, and duration of individual drug classes were not available. The exact day from admission to phenotyping was also not retained as a standardized analytic variable, so comparability by hospitalization day cannot be verified. Because every participant was receiving antihypertensive therapy, the recorded number of drug classes may partly reflect treatment intensity and underlying disease severity; however, without dose, adherence, timing, duration, and out-of-hospital BP data, class count cannot establish treatment adequacy or resistant hypertension. Accordingly, the study supports an association with the standardized in-hospital BP phenotype and should not be interpreted as validating capillaroscopy against longitudinal or ambulatory BP control.

Fourth, formal intra- and inter-observer reproducibility testing for capillaroscopy and carotid ultrasonography was not performed within this cohort, so the precision and repeatability of these operator-dependent measurements could not be quantified. Local coefficients of variation, intraclass correlation coefficients, standard errors of measurement, and limits of agreement are therefore unavailable, and the observed between-group differences cannot be formally benchmarked against cohort-specific measurement error. Likewise, the composite 0–10 microvascular score was constructed locally and has not been externally validated. Its cut-points were specified before outcome modeling and were not optimized against BP status or cIMT, which reduces but does not eliminate the risk inherent to a data-distribution-informed composite. Calibration, reproducibility, transportability, and incremental value over individual capillaroscopic measures remain to be demonstrated in an independent cohort.

Fifth, the database contained mean cIMT but did not contain a detailed focal plaque phenotype. Diffuse cIMT and plaque should not be conflated, and the ≥0.90 mm analysis was deliberately secondary. Sixth, only 38 participants met the ≥0.90 mm threshold, limiting the number of covariates that could be included in the corresponding logistic model and increasing uncertainty around subgroup analyses.

Seventh, residual confounding is unavoidable. Age, diabetes, kidney disease, smoking, lipid exposure, medications, and other unmeasured factors can influence one or more vascular measures. Known systemic sclerosis, other connective-tissue disease, and Raynaud phenomenon were exclusion criteria, but the analytic dataset did not contain a protocol-mandated rheumatologic serology panel; occult or previously undiagnosed rheumatologic disease therefore cannot be completely excluded. Diabetes status was adjusted for, but diabetes duration and standardized microvascular-complication variables were unavailable, so residual confounding by diabetic microangiopathy remains possible. In addition, hs-CRP was the only systemic inflammatory biomarker available; IL-6, TNF-α, endothelial-activation markers, and oxidative-stress biomarkers were not measured. The absence of an hs-CRP difference between BP-phenotype groups therefore does not exclude inflammatory or oxidative mechanisms. Although key covariates were adjusted for, the attenuation of the continuous cIMT association in the expanded model demonstrates that the observed cIMT association is modest and sensitive to model specification.

Eighth, multiple vascular comparisons increase the probability of chance findings. FDR correction was used for the primary correlation matrix, but descriptive group comparisons were not multiplicity-adjusted and should be interpreted as phenotype characterization rather than a set of independent confirmatory hypotheses. Ninth, the cohort lacks prospective cardiovascular, renal, or cognitive outcomes and an external validation sample, so incremental prognostic utility cannot be claimed.

Finally, an additional potential selection mechanism relates to HbA1c testing. Because hospital reagents were temporarily unavailable, HbA1c required external processing in a private laboratory and the cost was borne by the patients. All 217 participants in the analytic cohort had completed this component, but the source database did not retain the total number of hypertensive admissions, the number who began extended phenotyping, the number who failed to complete the bundle, or standardized reasons for noncompletion. The principal multivariable models did not include HbA1c, so the reported primary adjusted associations are not mathematically dependent on that assay. Nevertheless, willingness or ability to complete externally processed testing may have influenced representation in the fully phenotyped cohort. Because a screening/noncompletion log was not retained, this possible selection mechanism cannot be quantified and a formal recruitment flow diagram cannot be reconstructed without inventing data.

### 4.11. Future Directions

The next step should be a prospective, preferably multicenter study using consecutive screening, a prospectively registered analysis plan, ABPM or standardized home BP monitoring, medication-adherence assessment, quantitative carotid plaque characterization, and longitudinal cardiovascular and renal outcomes. External validation of the locally constructed microvascular score should be performed with the present component definitions, thresholds, and weights locked before analysis and applied unchanged to an independent cohort. The validation protocol should include blinded central or duplicate image reading, a prespecified reproducibility subset with intra- and inter-observer ICCs, coefficients of variation and agreement analyses, assessment of score distribution and construct validity, and comparison with key individual components such as capillary density. Transportability should be examined across clinically relevant strata including sex, diabetes, and kidney disease, while avoiding outcome-driven re-optimization in the validation sample. If the score is later considered for classification or prognostic use, calibration, discrimination, incremental value over conventional risk factors, and clinically relevant decision thresholds should be evaluated in a separate stage. Serial capillaroscopy could determine whether microvascular structure or flow changes with sustained BP improvement and whether changes parallel FMD, RHI, PWV, albuminuria, or carotid progression. Automated image analysis and machine learning-assisted quantification may ultimately improve reproducibility, but any algorithm should be validated against transparent, clinically interpretable capillaroscopic features rather than used as a black-box substitute for standardized acquisition.

## 5. Conclusions

Among 217 fully phenotyped treated adults with arterial hypertension, greater nailfold microvascular abnormality burden was associated with the uncontrolled standardized in-hospital BP phenotype and co-segregated with an adverse systemic vascular phenotype comprising thicker carotid walls, impaired endothelial function, increased arterial stiffness, albuminuria, lower renal function, and poorer cognitive performance. The microvascular score remained associated with the uncontrolled standardized in-hospital BP phenotype after clinical adjustment and showed a modest association with continuous cIMT in the primary model; however, this association attenuated and was no longer statistically significant in the expanded sensitivity model. Accordingly, the cIMT finding should be regarded as modest, model-sensitive, and hypothesis-generating rather than as evidence of a fully independent relationship. Critically, the BP association applies only to the protocolized in-hospital measurement phenotype studied here; it does not establish 24 h, home, or long-term BP control and cannot distinguish sustained uncontrolled hypertension from white-coat or masked uncontrolled BP patterns. These findings support nailfold capillaroscopy as a promising complementary research tool for integrated vascular phenotyping, not as a replacement for ABPM/home BP assessment, established carotid imaging, or guideline-based HMOD evaluation. Prospective external validation with consecutive recruitment, locked score definitions, formal measurement reproducibility assessment, and independent validation is required before diagnostic, prognostic, or clinical implementation.

## Figures and Tables

**Table 1:** Clinical, laboratory, vascular, and capillaroscopic characteristics of the study population.

Characteristic	Overall Cohort (*n* = 217)
Age, years	61.3 ± 9.9
Male sex	110 (50.7%)
Education, years	12.8 ± 2.8
BMI, kg/m^2^	29.3 ± 4.0
Hypertension duration, years	10.8 ± 6.7
SBP, mmHg	142.6 ± 13.3
DBP, mmHg	82.5 ± 7.9
Mean arterial pressure, mmHg	102.6 ± 6.5
Pulse pressure, mmHg	60.1 ± 16.2
Uncontrolled in-hospital BP phenotype	145 (66.8%)
Antihypertensive classes	2 [2–3]
ACE inhibitor/ARB	174 (80.2%)
Calcium-channel blocker	124 (57.1%)
Diuretic	88 (40.6%)
Beta-blocker	89 (41.0%)
Statin therapy	166 (76.5%)
Active smoking	47 (21.7%)
Former smoking	83 (38.2%)
Diabetes mellitus	53 (24.4%)
Dyslipidemia	129 (59.4%)
Chronic kidney disease	31 (14.3%)
Established cardiovascular disease	41 (18.9%)
LDL-C, mg/dL	117.1 ± 26.4
HbA1c, %	5.89 ± 0.89
eGFR, mL/min/1.73 m^2^	81.7 ± 14.9
UACR, mg/g	16.0 [9.0–31.4]
hs-CRP, mg/L	2.22 [1.25–3.13]
FMD, %	6.16 ± 1.34
RHI	1.83 ± 0.28
PWV, m/s	9.80 ± 1.33
Capillary density, /mm	8.59 ± 0.74
Erythrocyte velocity, mm/s	1.01 ± 0.13
Apical width, μm	28.95 ± 2.84
Tortuous capillaries, %	20.31 ± 8.65
Dilated loops, %	7.05 ± 4.62
Microhemorrhages	29 (13.4%)
Avascular areas	30 (13.8%)
Ramified capillaries	24 (11.1%)
Microvascular score (0–10)	3 [1–4]
MoCA score	26.46 ± 2.22
Cognitive impairment	78 (35.9%)
Mean cIMT, mm	0.776 ± 0.112
cIMT ≥ 0.90 mm	38 (17.5%)

Values are mean ± SD, median [IQR], or *n* (%), as appropriate. ACE, angiotensin-converting enzyme; ARB, angiotensin receptor blocker; BMI, body mass index; BP, blood pressure; cIMT, carotid intima-media thickness; DBP, diastolic blood pressure; eGFR, estimated glomerular filtration rate; FMD, flow-mediated dilation; HbA1c, glycated hemoglobin; hs-CRP, high-sensitivity C-reactive protein; IQR, interquartile range; LDL-C, low-density lipoprotein cholesterol; MoCA, Montreal Cognitive Assessment; PWV, pulse wave velocity; RHI, reactive hyperemia index; SBP, systolic blood pressure; SD, standard deviation; UACR, urinary albumin-to-creatinine ratio.

**Table 2:** Clinical and vascular phenotype according to the standardized in-hospital BP phenotype.

Variable	Controlled In-Hospital BP Phenotype (*n* = 72)	Uncontrolled In-Hospital BP Phenotype (*n* = 145)	*p* Value
Age, years	58.03 ± 8.73	62.87 ± 10.06	<0.001
BMI, kg/m^2^	29.33 ± 3.68	29.31 ± 4.10	0.971
Hypertension duration, years	10.20 ± 6.62	11.07 ± 6.79	0.368
FMD, %	6.53 ± 1.15	5.98 ± 1.40	0.002
RHI	1.95 ± 0.27	1.77 ± 0.27	<0.001
PWV, m/s	9.27 ± 1.32	10.06 ± 1.26	<0.001
Capillary density, /mm	8.90 ± 0.65	8.44 ± 0.74	<0.001
Erythrocyte velocity, mm/s	1.06 ± 0.12	0.99 ± 0.13	<0.001
Apical width, μm	28.74 ± 2.84	29.06 ± 2.84	0.429
Tortuous capillaries, %	16.49 ± 7.35	22.22 ± 8.64	<0.001
Dilated loops, %	6.00 ± 4.51	7.58 ± 4.60	0.017
Microvascular score	2 [1–3]	3 [2–4]	<0.001
UACR, mg/g	10.9 [5.7–19.6]	20.7 [10.3–43.0]	<0.001
hs-CRP, mg/L	2.4 [1.4–3.1]	2.0 [1.2–3.1]	0.149
MoCA	27.29 ± 1.98	26.04 ± 2.22	<0.001
cIMT, mm	0.74 ± 0.10	0.79 ± 0.12	0.001
Microhemorrhages	6 (8.3%)	23 (15.9%)	0.125
Avascular areas	4 (5.6%)	26 (17.9%)	0.013
Ramified capillaries	7 (9.7%)	17 (11.7%)	0.658
Cognitive impairment	17 (23.6%)	61 (42.1%)	0.008

Continuous variables were compared with Welch *t*-tests or Mann–Whitney U tests as appropriate; categorical variables with chi-square or Fisher exact tests. *p* values are unadjusted descriptive comparisons.

**Table 3:** Vascular and end-organ phenotype according to the exploratory cIMT threshold.

Variable	cIMT < 0.90 mm (*n* = 179)	cIMT ≥ 0.90 mm (*n* = 38)	*p* Value
Age, years	59.01 ± 8.94	71.89 ± 6.71	<0.001
BMI, kg/m^2^	29.35 ± 3.97	29.14 ± 3.93	0.766
Hypertension duration, years	10.05 ± 6.53	14.20 ± 6.67	<0.001
FMD, %	6.39 ± 1.25	5.05 ± 1.20	<0.001
RHI	1.88 ± 0.27	1.60 ± 0.21	<0.001
PWV, m/s	9.52 ± 1.20	11.08 ± 1.15	<0.001
Capillary density, /mm	8.69 ± 0.71	8.14 ± 0.73	<0.001
Erythrocyte velocity, mm/s	1.03 ± 0.12	0.91 ± 0.14	<0.001
Apical width, μm	28.70 ± 2.73	30.17 ± 3.08	0.009
Tortuous capillaries, %	18.78 ± 8.20	27.54 ± 6.99	<0.001
Dilated loops, %	6.51 ± 4.53	9.62 ± 4.23	<0.001
Microvascular score	2 [1–3]	5 [4–5]	<0.001
UACR, mg/g	13.9 [8.0–29.9]	23.1 [12.9–53.9]	0.001
hs-CRP, mg/L	2.1 [1.2–3.0]	2.7 [1.8–3.4]	0.031
MoCA	26.84 ± 2.12	24.66 ± 1.81	<0.001
cIMT, mm	0.74 ± 0.08	0.95 ± 0.05	<0.001
Microhemorrhages	21 (11.7%)	8 (21.1%)	0.125
Avascular areas	16 (8.9%)	14 (36.8%)	<0.001
Ramified capillaries	18 (10.1%)	6 (15.8%)	0.390
Cognitive impairment	50 (27.9%)	28 (73.7%)	<0.001

The 0.90 mm threshold was used as an exploratory historical marker of increased carotid wall thickness and was not considered equivalent to focal carotid plaque.

**Table 4:** Primary multivariable association models.

Outcome/Predictor	Effect Estimate	95% CI	*p* Value	Interpretation
Uncontrolled in-hospital BP phenotype: microvascular score, per 1 point	OR 1.70	1.31–2.20	<0.001	Primary adjusted logistic association
Uncontrolled in-hospital BP phenotype: age, per 10 years	OR 1.05	0.68–1.63	0.822	Adjusted covariate
Uncontrolled in-hospital BP phenotype: male sex	OR 1.31	0.70–2.44	0.402	Adjusted covariate
Uncontrolled in-hospital BP phenotype: BMI, per 5 kg/m^2^	OR 0.86	0.58–1.27	0.438	Adjusted covariate
Uncontrolled in-hospital BP phenotype: HTN duration, per 5 years	OR 1.00	0.80–1.26	0.985	Adjusted covariate
Uncontrolled in-hospital BP phenotype: diabetes	OR 1.05	0.44–2.49	0.908	Adjusted covariate
Uncontrolled in-hospital BP phenotype: active smoking	OR 1.14	0.51–2.52	0.747	Adjusted covariate
Uncontrolled in-hospital BP phenotype: eGFR, per 10 mL/min/1.73 m^2^	OR 0.92	0.69–1.21	0.538	Adjusted covariate
Continuous cIMT: microvascular score, per 1 point	β +0.0076 mm	0.0004 to 0.0147	0.040	Primary robust linear association
Continuous cIMT: age, per 10 years	β +0.0547 mm	0.0420 to 0.0675	<0.001	Primary robust linear model
Continuous cIMT: HTN duration, per 5 years	β +0.0104 mm	0.0017 to 0.0190	0.019	Primary robust linear model
Continuous cIMT: diabetes	β +0.0399 mm	0.0130 to 0.0667	0.004	Primary robust linear model
Continuous cIMT: LDL-C, per 10 mg/dL	β +0.0054 mm	0.0014 to 0.0095	0.009	Primary robust linear model
Continuous cIMT: eGFR, per 10 mL/min/1.73 m^2^	β −0.0089 mm	−0.0177 to −0.0001	0.049	Primary robust linear model
Continuous cIMT: microvascular score, expanded sensitivity model	β +0.0058 mm	−0.0018 to 0.0134	0.138	Attenuated after adding BMI, SBP, smoking

HTN, hypertension; OR, odds ratio. The uncontrolled in-hospital BP phenotype model included age, sex, BMI, hypertension duration, diabetes, active smoking, eGFR, and microvascular score. The primary continuous-cIMT model included age, sex, hypertension duration, diabetes, LDL-C, eGFR, and microvascular score; Figure 4B displays the complete coefficient set, including male sex; this table reports the principal numeric estimates. The expanded sensitivity model additionally included BMI, SBP, and active smoking. HC3 robust standard errors were used for linear cIMT models.

## Data Availability

De-identified patient-level data supporting the findings are not publicly posted because of institutional privacy and ethics constraints. They may be made available by the corresponding author upon reasonable request, subject to Ethics Council approval, institutional data-sharing policies, and applicable privacy requirements.

## Supplementary Materials

The following supporting information can be downloaded at: https://www.mdpi.com/article/10.3390/jcm15187097/s1, Table S1: Composite microvascular score—component definitions and point allocation; Table S2: Spearman correlations with mean cIMT; Table S3: Expanded sensitivity model for continuous mean cIMT; Table S4: Exploratory logistic model for cIMT ≥ 0.90 mm; Table S5: Collinearity and interaction diagnostics.

## Abbreviations

The following abbreviations are used in this manuscript:

ABPM	ambulatory blood-pressure monitoring
ACE	angiotensin-converting enzyme
ARB	angiotensin receptor blocker
BMI	body mass index
BP	blood pressure
CI	confidence interval
cIMT	carotid intima-media thickness
CKD-EPI	Chronic Kidney Disease Epidemiology Collaboration
DBP	diastolic blood pressure
eGFR	estimated glomerular filtration rate
FDR	false-discovery rate
FMD	flow-mediated dilation
HbA1c	glycated hemoglobin
HMOD	hypertension-mediated organ damage
hs-CRP	high-sensitivity C-reactive protein
HTN	hypertension
IQR	interquartile range
LDL-C	low-density lipoprotein cholesterol
MoCA	Montreal Cognitive Assessment
OR	odds ratio
PWV	pulse wave velocity
RHI	reactive hyperemia index
SBP	systolic blood pressure
SD	standard deviation
UACR	urinary albumin-to-creatinine ratio
