# Supplementary material for: Nailfold Microvascular Abnormalities Are Associated with the Standardized In-Hospital Blood-Pressure Phenotype and Carotid Wall Thickness in Treated Hypertension: An Integrated Vascular Phenotyping Study
Source: J Clin Med. 2026 Sep 13;15(18):7097. doi: 10.3390/jcm15187097 (PMC13607206; doi:10.3390/jcm15187097)
Supplement: Supplementary file 1 [file jcm-15-07097-s001.zip › jcm-4552546-supplementary.pdf]

Supplementary Material

Nailfold Microvascular Dysfunction Is Associated with In-Hospital Blood-Pressure Control and Carotid Wall Remodeling in Treated Hypertension: An Integrated Vascular Phenotyping Study

Table S1. Composite microvascular score – component definitions and point allocation

Component	0 points	1 point	2 points
Capillary density (capillaries/mm)	≥9.0	8.0–8.9	<8.0
Erythrocyte velocity (mm/s)	≥1.00	0.85–0.99	<0.85
Tortuous capillaries (%)	<20	20–29.9	≥30
Dilated apical loops (%)	<10	10–19.9	≥20
Microhemorrhages	Absent	Present	Not applicable
Avascular areas	Absent	Present	Not applicable

The six component scores are summed to yield a composite score ranging from 0 to 10 points, with higher values indicating a greater burden of nailfold microvascular abnormality. Continuous features contribute 0–2 points and the two binary morphological abnormalities contribute 0–1 point each. Component cut-points were specified before outcome modelling and were informed by the distribution of the capillaroscopic variables solely to create ordinal severity bands. Blood-pressure-control status, cIMT, receiver-operating-characteristic analysis, p values, or any other outcome-based optimization were not used to select the cut-points or component weights. No diagnostic cut-off for the total score was defined. The score was not used to define study eligibility or either study outcome. Because it is a locally constructed, unvalidated research index, its calibration, reproducibility, transportability, and incremental value require independent external validation. The observed total-score range in the present cohort was 0 to 8 points.

Table S2. Spearman correlations with mean cIMT

Variable	Spearman ρ	Raw p	FDR-adjusted p
Microvascular score	0.536	<0.001	<0.001
Capillary density	−0.384	<0.001	<0.001
Erythrocyte velocity	−0.455	<0.001	<0.001
Apical width	0.211	0.002	0.002
Tortuous capillaries	0.383	<0.001	<0.001
Dilated loops	0.363	<0.001	<0.001
FMD	−0.373	<0.001	<0.001
RHI	−0.531	<0.001	<0.001
PWV	0.540	<0.001	<0.001
UACR	0.302	<0.001	<0.001
eGFR	−0.546	<0.001	<0.001
MoCA	−0.489	<0.001	<0.001
Age	0.686	<0.001	<0.001
SBP	0.315	<0.001	<0.001
LDL-C	0.197	0.004	0.004

The Benjamini–Hochberg procedure was applied across the 15 correlations reported in this table. cIMT, carotid intima-media thickness; eGFR, estimated glomerular filtration rate; FDR, false-discovery rate; FMD, flow-

mediated dilation; LDL-C, low-density lipoprotein cholesterol; MoCA, Montreal Cognitive Assessment; PWV, pulse wave velocity; RHI, reactive hyperemia index; SBP, systolic blood pressure; UACR, urinary albumin-to-creatinine ratio.

Table S3. Expanded sensitivity model for continuous mean cIMT

Predictor	β (mm)	Robust SE	95% CI	p value
Age, per 10 years	0.0559	0.0067	0.0428 to 0.0690	<0.001
Male sex	0.0120	0.0110	−0.0096 to 0.0335	0.276
BMI, per 5 kg/m²	−0.0036	0.0066	−0.0165 to 0.0093	0.583
Hypertension duration, per 5 years	0.0105	0.0045	0.0017 to 0.0192	0.019
SBP, per 10 mmHg	0.0027	0.0049	−0.0069 to 0.0123	0.581
LDL-C, per 10 mg/dL	0.0058	0.0021	0.0017 to 0.0099	0.006
Diabetes mellitus	0.0414	0.0141	0.0138 to 0.0690	0.003
Active smoking	0.0225	0.0132	−0.0034 to 0.0484	0.089
eGFR, per 10 mL/min/1.73 m²	−0.0087	0.0047	−0.0179 to 0.0005	0.064
Microvascular score, per 1 point	0.0058	0.0039	−0.0018 to 0.0134	0.138

Model $R^2 = 0.580$. HC3 heteroscedasticity-consistent standard errors were used. This maximal model adds BMI, current SBP, and active smoking to the primary clinical adjustment set. BMI, body mass index; CI, confidence interval; SE, standard error.

Table S4. Exploratory logistic model for cIMT ≥0.90 mm

Predictor	Adjusted OR	95% CI	p value
Age, per 10 years	5.31	2.56–10.99	<0.001
Male sex	0.57	0.23–1.45	0.238
Hypertension duration, per 5 years	1.69	1.16–2.47	0.007
Diabetes mellitus	2.32	0.87–6.21	0.093
Microvascular score, per 1 point	1.48	1.07–2.04	0.017

Only 38 participants met the cIMT ≥0.90-mm endpoint; the model was therefore deliberately parsimonious and its results are hypothesis-generating. OR, odds ratio.

Table S5. Collinearity and interaction diagnostics

Primary continuous-cIMT predictor	Variance-inflation factor
Age, per 10 years	1.93
Male sex	1.04
Hypertension duration, per 5 years	1.04
Diabetes mellitus	1.25
LDL-C, per 10 mg/dL	1.08

Primary continuous-cIMT predictor	Variance-inflation factor
eGFR, per 10 mL/min/1.73 m^2	1.78
Microvascular score, per 1 point	1.72

All variance-inflation factors were below 2, providing no evidence of problematic multicollinearity in the primary cIMT model.

Exploratory interaction p values

Interaction	p value
Microvascular score × sex → uncontrolled hypertension	0.784
Microvascular score × diabetes → uncontrolled hypertension	0.770
Microvascular score × sex → continuous cIMT	0.057
Microvascular score × diabetes → continuous cIMT	0.494

Interaction analyses were exploratory; the study was not specifically powered to detect subgroup effect modification.
